# Overcoming the challenges of imaging patients with metabolic syndrome

**DOI:** 10.1590/0100-3984.2019.52.1e1

**Published:** 2019

**Authors:** Michael A. Ohliger, Antonio C. Westphalen

**Affiliations:** 1 MD PhD, Department of Radiology and Biomedical Imaging, University of California San Francisco (UCSF), Zuckerberg San Francisco General Hospital and Trauma Center, San Francisco, CA, USA. https://orcid.org/0000-0001-6878-8189.; 2 MD PhD, Department of Radiology and Biomedical Imaging, and Urology, University of California San Francisco (UCSF), San Francisco, CA, USA. Email: AntonioCarlos.Westphalen@ucsf.edu. https://orcid.org/0000-0003-0071-4989.

Obesity and metabolic syndrome are well recognized public health issues that affect the populations of many countries. Any effort aimed at addressing the problem should be praised. The work of Pescatori et al.^(^1^)^ published in this issue of **Radiologia Brasileira** evaluated the inter- and intra-observer variability of visceral fat measurements made on T1- and T2-weighted magnetic resonance (MR) images and compared these measurements to those obtained using computed tomography (CT) images. Thirty-one patients who had a CT and MR scan done within 90 days of each other were included in this study. Unenhanced CT and T1- and T2-weighted MR images were copied into a separate workstation loaded with OsiriX. Two readers independently measured the area of visceral fat on single slice acquired at the level of the umbilicus utilizing a semi-automated tool. According to their results, visceral fat measurements obtained with MR images are accurate when compared to CT scan measurements. Furthermore, all methods have high inter- and intra-observer reproducibility, though T1-weighted images may be less reproducible.

A critical analysis of any published study is always recommended for understanding of its results and conclusions. With retrospective studies, for example, we must examine the effect potential biases might have had on the results presented. In addition, the evaluation of any diagnostic test must always consider other existing alternatives, which may have advantages (e.g. greater availability, high accuracy, lower cost, or better safety profile) that may favor its use.

As the authors acknowledge, images obtained at the level of the umbilicus (or at any other location) may not be comparable at two different time points because of changes in position of bowel, differences in breathing, or even changes in weight. It would not be unexpected for some patients to lose or gain enough fat in 90 days to impact the results of this study, particularly oncologic patients (n = 14/31) and patients with inflammatory bowel disease (n = 5/31). It would have been interesting, therefore, if the authors had reported the patient weight or body mass index (BMI) at the time of scanning. Another approach to account for these possible variations is to measure visceral fat in more than one slice, as the authors recognize. This would indeed add complexity to the process, but would have minimized the impact of existing variations.

Clinical tools are not flawless, and certainly not as accurate as cross-sectional imaging to determine the volume of visceral fat. Yet, these are methods that can be quickly utilized during any office visit. BMI, for example, is an established method to define obesity and is reliable enough to screen patients who might be at higher risk of metabolic syndrome and its associated ailments. The goal of imaging, therefore, should not be to replace these tools, but to improve upon them. Cross-sectional imaging is able to provide us with an accurate estimate of visceral fat area or volume, but it is usually not practical to obtain these measurements routinely. It is clear that a simpler method of estimating both visceral and total fat is desirable. Furthermore, if one considers cost and time, it is unlikely that clinicians would request a dedicate scan, even if a limited one, to assess the extent of visceral fat. Yet, the assessment of visceral fat may have a role in a comprehensive scan that investigates a more extensive set of imaging biomarkers, for example liver stiffness, fat, and inflammation.

If radiologists want to disseminate imaging biomarkers, two barriers must be overcome: limited integration of proprietary software to the workflow and lack of standardization of measurements. Until the various PACS platforms permit the easy integration of vendor-neutral plugins, the need for separate workstations and the inability to perform the same calculations across centers will hinder these efforts. Lack of a set of standards for calculating quantitative imaging parameters makes the matter worse. The main imaging societies are pursuing these initiatives, but meanwhile academicians who use "home-built" tools in research should be encouraged to make those tools freely available to others. The use of a widely available open tool as OSIRIX is a step in the right direction, as it allows other investigators to reproduce the work presented in this issue.

Just as clinicians regularly weigh patients and calculate BMI, radiologists might be able to do estimate patients' risk on routine imaging. Radiologists must continue to improve and validate the available imaging tools so that these can be easily applied to our routine scans. The results of this study provide support for further exploration of MR imaging for quantification of visceral fat, perhaps combined with other mechanisms, e.g. fully automated segmentation implemented into the scanner's console or PACS. As important, though, is the development of large-scale outcomes research to determine the association between baseline and changes of visceral fat over time with hard outcomes such as death.
